# CLINICIAN AND STAFF PERSPECTIVES IN TRANSITIONS OF CARE FOR DUAL-USE VETERANS LIVING WITH DEMENTIA

**DOI:** 10.1093/geroni/igad104.2402

**Published:** 2023-12-21

**Authors:** Roman Ayele, Karen Albright, Catherine Battaglia, Cari Levy

**Affiliations:** VA Eastern Colorado Health Care System, Aurora, Colorado, United States; VA Eastern Colorado Healthcare System, Aurora, Colorado, United States; VA Eastern Colorado Health Care System, Aurora, Colorado, United States; University of Colorado, Denver, Colorado, United States

## Abstract

The Veterans Administration (VA) has partnered with non-VA hospitals to improve access to care. However, older Veterans living with dementia (VLWD) who use both VA and non-VA care may experience gaps in care, placing them at risk of adverse health outcomes during transitions. This research aims to understand the barriers and facilitators in transitions of care for VLWD following non-VA hospitalization. We conducted 23 interviews with VA and non-VA clinicians involved in transitions of care for VLWD. Snowball sampling was used to identify participants, and rapid analysis identified themes. VA Participants faced significant challenges in receiving timely discharge communication from non-VA stakeholders, relying on VLWD, who are poor historians, for information about their non-VA hospitalization. Inadequate staffing for care coordination was also a barrier to timely care, resulting in frustration for VLWD and their families when tests had to be redone due to a lack of appropriate documentation from non-VA hospitals. Both VA and non-VA stakeholders experienced similar care coordination challenges for VLWD, their families, and clinicians. Non-VA stakeholders described the VA as a “black box” due to a lack of communication and collaboration. However, stakeholders expressed a desire to provide quality care, improve care coordination, and give timely care to veterans. These findings highlight the barriers experienced by VA and non-VA stakeholders and provide opportunities for improvement in coordinating care for VLWD. Implementing care coordination programs and improving communication and collaboration between VA and non-VA stakeholders can improve the quality of care for VLWD and their families during transitions.

